# Potential for Plant Growth Promotion of Rhizobacteria Associated with *Salicornia* Growing in Tunisian Hypersaline Soils

**DOI:** 10.1155/2013/248078

**Published:** 2013-05-28

**Authors:** Francesca Mapelli, Ramona Marasco, Eleonora Rolli, Marta Barbato, Hanene Cherif, Amel Guesmi, Imen Ouzari, Daniele Daffonchio, Sara Borin

**Affiliations:** ^1^DeFENS, Department of Food, Environment and Nutritional Sciences (DeFENS), University of Milan, Via Celoria 2, 20133 Milan, Italy; ^2^Laboratory of Microorganisms and Active Biomolecules, University of Tunis El Manar, Campus Universitaire, 2092 Tunis, Tunisia

## Abstract

Soil salinity and drought are among the environmental stresses that most severely affect plant growth and production around the world. In this study the rhizospheres of *Salicornia* plants and bulk soils were collected from *Sebkhet* and *Chott* hypersaline ecosystems in Tunisia. Depiction of bacterial microbiome composition by Denaturing Gradient Gel Electrophoresis unveiled the occurrence of a high bacterial diversity associated with *Salicornia* root system. A large collection of 475 halophilic and halotolerant bacteria was established from *Salicornia* rhizosphere and the surrounding bulk soil, and the bacteria were characterized for the resistance to temperature, osmotic and saline stresses, and plant growth promotion (PGP) features. Twenty *Halomonas* strains showed resistance to a wide set of abiotic stresses and were able to perform different PGP activities *in vitro* at 5% NaCl, including ammonia and indole-3-acetic acid production, phosphate solubilisation, and potential nitrogen fixation. By using a *gfp*-labelled strain it was possible to demonstrate that *Halomonas* is capable of successfully colonising *Salicornia* roots in the laboratory conditions. Our results indicated that the culturable halophilic/halotolerant bacteria inhabiting salty and arid ecosystems have a potential to contribute to promoting plant growth under the harsh salinity and drought conditions. These halophilic/halotolerant strains could be exploited in biofertilizer formulates to sustain crop production in degraded and arid lands.

## 1. Introduction

The influence of microbes on plant fitness has been recognized both in conventional and extreme habitats, where the ability of rhizobacteria to facilitate plant adaptation and promote growth and productivity has been reported [1–6]. Root-associated bacteria can promote plant growth by direct and indirect mechanisms, the former including nutrient fixation and solubilisation and phytohormones synthesis. Indirect activities include biocontrol, the ability to reduce or avoid the harmful effects of phytopathogens. Both the host plant and its associated microbiome gain an evolutionary advantage to survive under harsh conditions by establishing tight interplays.

Among abiotic stresses soil salinity is one of the strongest factors affecting plant growth and yield [7]. Conditions of high salt concentrations in the soil are very frequent in arid and semiarid regions on Earth, where different halophytic species can be found. Halophytes have been proposed as key players for saline soils reclamation [8], phytoremediation of hydrocarbon and heavy metals polluted saline soils [9, 10], and forage and oil seed production [11, 12]. *Salicornia* (Chenopodiaceae) is a subcosmopolitan plant genus comprising annual species strictly occurring in salty environments and widespread in several countries, including those of the Mediterranean basin. *Salicornia* densely colonises different areas of southern Tunisia, including *Sebkhet* and *Chott* ecosystems, dominated by extreme values of aridity and soil salinity. Intense evaporation rates render *Sebkhet* and *Chott* as dry salt lakes which are inhospitable for most of the organisms.

The manipulation of natural resources to increase plant productivity in lands traditionally considered unsuitable for agriculture is a challenging but necessary task, in the light of the increasing world population and the need for food production [13]. The efforts which aimed to the production of salt-resistant crops include conventional breeding, marker-assisted selection, and the creation of transgenic plants and are nowadays focusing also on the halophyte potential to guarantee a suitable food production in a salinized planet [14]. Different works in the last years highlighted the importance of plant growth promoting bacteria in facilitating salt tolerance in plants devoted to food production [3, 7, 15, 16], and few reports emphasized the role of PGP bacteria associated with *Salicornia* spp. [17–22]. The investigation of the rhizobacterial community associated to plants naturally adapted to cope with extreme saline conditions might lead to several knowledge outputs: (i) the understanding of the plant-microbe interaction under saline conditions, (ii) definition of the mechanisms underlying plant growth with promotion under the salinity stress, and (iii) identification of bacterial strains to design biological fertilizers exploitable for agriculture in arid and saline lands. To achieve the best results in terms of plant growth promotion under salinity and drought stress it is essential to focus on the fraction of the culturable bacteria that is able to thrive under these specific conditions. Therefore, the aims of this work were (i) the isolation of halophilic/halotolerant bacteria from *Salicornia* rhizosphere and bulk soils collected in hypersaline ecosystems in southern Tunisia, (ii) the characterization of their resistance to abiotic stresses and their plant growth promoting (PGP) potential, and (iii) the description of taxonomic diversity of both the halophilic/halotolerant culturable fraction and the whole bacterial microbiome inhabiting *Salicornia* rhizosphere and bulk soils.

## 2. Materials and Methods

### 2.1. Site Description, Soil Sampling, and Soil Characterization

The studied sites, named BDV4 (N 34°26′951′′; E 09°54′102′′), BDV11 (N 34°08′735′′; E 08°04′417′′), and BDV20 (N 33°57′252′′; E 08°24′508′′) corresponded, respectively, to *Sebkhet* El Naouel, *Chott* El Gharsa, and *Chott* El Jerid and were located in southern Tunisia.

Visual inspection of the sites identified *Salicornia* as the only present plant. The plants were identified according to the plant morphology as *S. strobilacea* [23, 24], a widespread plant in southern Tunisia.

Between the sites different conditions in respect of superficial salt crust presence (BDV11) or absence (BDV20) were observed (Table 1). Rhizospheric and bulk soils were sampled from triplicate specimens of *Salicornia* from sites BDV11 and BDV20. In the site BDV4 different microenvironments were identified, and a total of six *Salicornia* specimens were collected. In this site three sampled specimens were growing on salt crust covered soil (BDV4-S1, BDV4-S2, and BDV4-S3), and three sampled specimens were growing on a soil plot where salt crusts were absent (BDV4-S4, BDV4-S5, and BDV4-S6). Replicates of bulk soils were also sampled from the site BDV4 (presence of salt crust: BDV4-B1; absence of salt crust: BDV4-B4). Rhizospheric soil was defined as soil particles tightly adhering to roots (1–3 mm) after gently shaking. Bulk soil was collected as control about 2 m far from any vegetation. Rhizosphere and bulk soils will be, respectively, indicated in the text with the codes S and B. Soil samples were collected using sterile spoons and stored in sterile bags at −20°C for molecular analyses and at 4°C for microbiological isolation. Soil salinity was measured with a hand refractometer (Atago, Tokyo, Japan) after the extraction of pore water from approximately 2 g of soil.

### 2.2. Metagenome Extraction and 16S rRNA Amplification

DNA was extracted from 0.5 g of soil using the protocol established by Schbereiter-Gurtner et al. [25].

DNA was quantified using NanoDrop 1000 spectrophotometer (Thermo Scientific, Waltham, MA, USA).

Bacterial 16S rRNA gene fragments (~550 bp) were PCR amplified using primers 907R (3′-CCGTCAATTCCTTTGAGTTT-5′) and GC-357F (3′-CCTACGGGAGGCAGCAG-5′ with a 5′-end GC-clamp) targeting a portion of the 16S rRNA gene that include the hypervariable V3 regions [26]. PCR reactions were performed in a 50 *μ*L final volume containing 1X buffer, 2.5 mM MgCl_2_, 5% of DMSO, 0.12 mM of dNTPs mixture, 0.3 *μ*M of each primer, 1.5 U Taq polymerase, and 10 ng of template, applying the following thermic protocol: 94°C for 4 min, followed by 10 cycles of 94°C for 0.5 min, 61°C for 1 min, and 72°C for 1 min; followed by further 20 cycles of 94°C for 0.5 min, 56°C for 1 min, and 72°C for 1 min; and a final extension at 72°C for 7 min. Presence and length of PCR products were verified by electrophoresis in 1% w/v agarose gel prior to Denaturing Gradient Gel Electrophoresis (DGGE) analysis.

### 2.3. Denaturing Gradient Gel Electrophoresis

PCR products (~150 ng) were loaded in a 0.5 mm polyacrylamide gel (7% (w/v) acrylamide-bisacrylamide, 37.5 : 1) containing 40 to 60% urea-formamide denaturing gradient (100% corresponds to 7 M urea and 40% (vol/vol) formamide) according to the method described by Muyzer et al. [26]. The gels were run for 15 h at 60°C by applying a constant voltage of 90 V in 1X Tris-acetate-EDTA (TAE) buffer. After electrophoresis, the gels were stained for 30 min in 1X TAE buffer containing 1X SYBR Green (Molecular Probes, Leiden, the Netherlands) according to manufacturer's instructions and rinsed twice for 10 min with distilled water. Gels images were captured using a Gel Doc 2000 apparatus (Bio-Rad, Milan, Italy). The band patterns of DGGE gels were analysed using Image J software (available for free download at http://rsb.info.nih.gov/ij/) and Microsoft Excel XLSTAT software (Addinsoft Inc., New York, NY, USA) as previously described [5]. DGGE bands were excided from the gels with a sterile scalpel and eluted in 50 *μ*L of sterile Milli-Q water at 37°C for 4 hours. Subsequently, 8 *μ*L of eluted DNA was reamplified by PCR using primers 357F and 907R as described in the previous paragraph. Positive amplifications were partially sequenced by Macrogen Inc., Korea (http://www.macrogen.com/) using the primer 357F.

### 2.4. Bacteria Isolation

Rhizospheric and bulk soil triplicates were pooled prior to bacteria isolation, except in the case of the specimens BDV4-S4, BDV4-S5, and BDV4-S6, collected at site BDV4 in absence of surface salt crust, and processed separately. Thus, we compared the structure of culturable halotolerant/halophilic bacteria among the different study sites, and additionally the taxonomic diversity within the same site was evaluated by comparing the replicates. To determine the bacterial cell number, 1 g of rhizospheric and bulk soils collected at the different sites was shaken with 9 mL of sterile saline solution (0.9% NaCl). The suspensions were serially diluted and plated in triplicate on solidified R2A medium (Oxoid) enriched with 10 and 15% NaCl. After 1-week incubation at 30°C, the colony-forming unit (cfu) per gram was determined, and, for each sample, a number of colonies comprised between 8 and 42 per medium were randomly selected. The bacterial isolates were stored as 25% glycerol stocks at −80°C.

### 2.5. Genotypic Characterization and Identification

DNA extraction was performed on each isolated strain. The bacteria collection has been dereplicated through the application of 16S-23S rRNA Intergenic Transcribed Spacer-PCR (ITS-PCR) using ITS-F (3′-GTCGTAACAAGGTAGCCGTA-5′) and ITS-R (3′-CTACGGCTACCTTGTTACGA-5′) primers as previously described [5, 27]. PCR amplification was performed in 25 *μ*L reaction containing 1X buffer, 1.5 mM MgCl_2_, 0.12 mM of dNTPs mixture, 0.3 *μ*M of each primer, 1 U Taq polymerase, and 10 ng of template, applying the following thermic protocol: 94°C for 4 min, followed by 35 cycles of 94°C for 0.5 min, 55°C for 1 min, and 72°C for 2 min and a final extension at 72°C for 10 min. The ITS-PCR products were run on 2% agarose gels and stained with ethidium bromide. Gel images were captured using a Gel Doc 2000 apparatus (Bio-Rad, Milan, Italy) and ITS-fingerprinting profiles were visually analysed to cluster together the bacterial isolates showing the same band pattern. From each cluster, at least a representative strain has been selected for subsequent PGP characterization and genotypic identification through 16S rRNA gene sequencing. 16S rRNA amplification was performed by using the universal primers 27F (3′-AGAGTTTGATCMTGGCTCAG-5′) and 1492R (3′-CTACGGCTACCTTGTTACGA-5′) [28] and applying the same protocol of ITS-PCR. Partial 16S rRNA sequences were obtained from Macrogen Inc., Korea (http://www.macrogen.com/).

### 2.6. Nucleotide Sequence Analyses and Accession Numbers

Nucleotide sequences were edited in Chromas Lite 2.01 (http://www.technelysium.com.au) and subjected to BLAST search (http://blast.ncbi.nlm.nih.gov/Blast.cgi). The partial 16S rRNA gene sequences obtained from the bacterial isolates and the excised DGGE bands have been deposited in the EMBL, respectively, under accession numbers HF678717–HF678862 and HF678127–HF678194.

### 2.7. Resistance to Abiotic Stresses

Resistance to salt stress was assessed by growing the isolates at 30°C in R2A supplemented by different sodium chloride concentrations, ranging from 0 to 20% w/v. The ability to grow under osmotic stress was tested at 30°C by adding 5–20% of polyethylene glycol (PEG) to R2A broth medium. Finally, the capability to growth in a wide range of temperatures was verified by incubating the R2A plates at 4°, 42°, and 50°C. A control, consisting in a sterile plate or tube, was also run parallel to each experiment.

### 2.8. *In Vitro* Screening of Plant Growth Promoting Activities

Each isolate was grown as pure culture to evaluate its PGP features in suitable media enriched with 5% w/v NaCl. Only two isolates were not able to grow at 5% NaCl, and their PGP activities were tested at 10% NaCl. The production of indole-3-acetic acid was detected by the method described by Bric et al. [29]. The ability to solubilise insoluble phosphate compounds was estimated according to Ahmad et al. [30]. The ammonia synthesis assay was performed as recommended by Cappuccino and Sherman [31]. Protease activity was determined from clearing zones in skimmed milk agar according to Nielsen and Sørensen [32]. Atmospheric nitrogen fixation ability and ACC-deaminase activity were determined by the method of Penrose and Glick [33]. *nifH* gene detection has been performed by PCR test using the primer sets PolF (3′-TGCGAYCCSAARGCBGACTC-5′) and PolR (3′-ATSGCCATCATYTCRCCGGA-5′) [34]. PCR amplification was performed in 25 *μ*L reaction containing 1X buffer, 1.5 mM MgCl_2_, 0.12 mM of dNTPs mixture, 0.3 *μ*M of each primer, 1 U Taq polymerase, and 10 ng of template, applying the following thermic protocol: 94°C for 4 min, followed by 35 cycles of 94°C for 0.5 min, 55°C for 1 min, and 72°C for 2 min and a final extension at 72°C for 10 min.

### 2.9. Chromosomal *gfp*-Tagging of Halotolerant *Halomonas* Strains by Conjugation Procedure

To stably transform strains affiliated to the genus *Halomonas*, we adopted the method based on mini-Tn7 transposon system [35]. Briefly, the mobilisation of the *gfp*-harbouring fragment was achieved by a four-parental conjugation formed by a cellular suspension of 10^10^ cells of the strain to be transformed and 10^9^ cells for *E*. *coli* strains carrying helper, delivery, and mobilization plasmids [35]. To select for *gfp*-transformed cells, after the mating, the cellular suspension was plated in R2A medium supplemented with 10% NaCl and the required antibiotics. The *gfp*-labelling procedure was successful for a strain of *H. elongata*, as visualized by fluorescence microscopy.

### 2.10. *In Vitro* Bacterial Rhizocompetence Test

To evaluate *gfp*-labelled strain ability to adhere and potentially colonise plant root system, an *in vitro* assay was performed on two model plants: *Arabidopsis thaliana* and *Salicornia* plantlets collected in marine dune ecosystems in south Italy. After an overnight growth in liquid selective medium, bacterial cell concentration was microscopically evaluated, and a 10^8^ cell/mL suspension was prepared. *Salicornia* plant roots were dipped in MS salt half strength medium (SIGMA, Italy) supplemented with 2% NaCl and the prepared bacterial suspension. For *Arabidopsis* rhizocompetence test, NaCl addition was avoided since this plant is extremely salt stress-sensitive. After an overnight incubation (~16 h), plant roots were gently washed to remove no- or weakly-bound bacterial cells and observed under a confocal laser scanning microscope (Leica TCSNT). Images were acquired using Leica Confocal Software and analysed by using the MBF ImageJ software.

## 3. Results and Discussion

### 3.1. DGGE Analysis of the Bacterial Microbiome Inhabiting *Salicornia *Rhizosphere and Surrounding Bulk Soil

**Figure 1:**
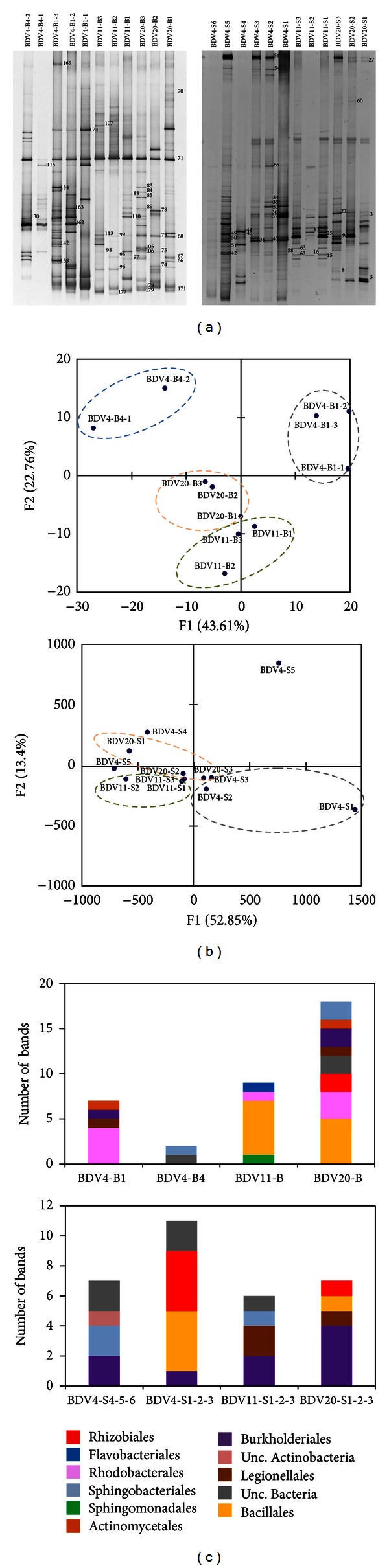
DGGE analysis performed on the bulk (left panel) and rhizospheric (right panel) soil bacterial community. (a) In the left panel, DGGE patterns of the bulk soils collected at sites BDV20, BDV11 and BDV4. In the right panel, DGGE patterns of the rhizospheric soils collected at sites BDV20, BDV11, and BDV4. The numbers represented the three analysed replicates. (b) Principal Component Analysis based on the DGGE profiles of the bacterial community inhabiting bulk (left) and rhizospheric (right) soil associated with *Salicornia* specimens. (c) Taxonomic identification of bacterial 16S rRNA sequences excised from DGGE bands cut from rhizospheric and bulk soil profiles.

The introduction of fingerprint-based analyses [26, 36, 37] turned into the application of cultivation-independent techniques as routine tools to depict the overall microbiome composition in environmental samples and to infer which factors influence the abundance and distribution of specific microbial taxa. Here, DGGE analysis of 16S rRNA gene was applied to provide a snapshot of both culturable and unculturable bacterial assemblages in *Salicornia* rhizosphere and the surrounding bulk soil not affected by the plant. Bulk soil samples from all sites were analysed in triplicate, except in BDV4-B4 sites where only duplicate samples were analysed due to the failing of PCR-DGGE amplification. Although the bulk soils collected from Tunisian *Sebkhet* and *Chott* were characterized by extreme dryness and salinity values, DGGE band profiles highlighted that a rich and diverse bacterial microbiome was present in all the samples (Figure 1(a), left panel). Principal Component Analysis (PCA) performed on the line plots derived from DGGE band profiles (Figure 1(b), left panel) indicated that bulk soils clustered according to the site of provenience. On axis 1, describing the 44% of the samples similarity, bulk soil samples were distributed according to the presence (stations BDV4-B1 and BDV11-B) or absence (stations BDV20-B and BDV4-B4) of salt crusts covering the soil surface. Salinity is known as one of the strongest abiotic factors influencing the assemblages of a huge variety of bacterial populations sheltered by the soil [38]. The factors shaping the composition of the bacterial community include also biotic interactions, and the role of root exudates in the selection of a peculiar microbiome is well known [39]. The DGGE pattern obtained from *Salicornia* rhizosphere samples was different from those observed in bulk soils (Figure 1(a)). According to DGGE fingerprints the bacterial communities of the rhizospheric soil triplicates collected at both sites BDV20 and BDV11 clustered together (Figure 1(a), right panel). Similarly, the rhizospheres collected from the salt crust covered soil at site BDV4 (BDV4-S1, 2, and 3) showed a high level of homogeneity. A high number of DGGE bands were observed in all the rhizospheres, with the exclusion of BDV4-S6 sample, probably affected by biases in PCR amplification. Overall, the rhizosphere of *Salicornia* was proved to be a habitat characterized by a highly rich bacterial community. Principal Component Analysis of the DGGE patterns (Figure 1(b), right panel) indicated, except for rhizosphere samples BDV4-S5 and BDV4-S1, a higher similarity among the rhizospheres collected from different stations than among the bulk soils suggesting that the rhizosphere acts as a selection factor that tend to uniform bacterial diversity independent from the soil type. Principal Component Analysis (Figure 1(b), right panel) pointed out the overall similarity of the bacterial communities hosted by the *Salicornia* rhizospheres collected in different sites. The even structure of the rhizosphere bacterial community confirmed the importance of plant inputs in the selection of specific bacterial taxa associated with the roots, a well-known phenomenon generally reported as “rhizosphere effect.” Despite that, the occurrence of a degree of variability within the bacterial microbiome associated with *Salicornia* specimens collected in different microenvironments within site BDV4 (related to presence and absence of salt crusts on soil surface) was perceived by DGGE analysis. This result confirmed that, besides the selection driven by the plant, also the environmental parameters play a role in shaping the rhizosphere bacterial microbiome.

According to DGGE band sequencing analyses, the prevalent taxonomic groups associated with *Salicornia *roots and bulk soils were Alpha-, Beta- and Gammaproteobacteria, Bacilli, and *Actinobacteria *(Figure 1(c)), as previously observed in the bacterial communities associated to different plant species (*Capsicum annuum*) growing in desert areas under water stress condition [5]. In addition, two different orders belonging to the Bacteroidetes phylum were retrieved, namely, Sphingobacteriales and Flavobacteriales, the latter exclusively present in the bulk soil (Figure 1(c)). Both in rhizospheric and bulk soils Beta- and Gammaproteobacteria and *Bacilli* were represented by only one taxonomic order, while *Alphaproteobacteria* composition differed in the two soil fractions. *Alphaproteobacteria* in rhizospheric soils were represented exclusively by Rhizobiales spp. while bulk soils were colonised by a more diverse bacterial community including, in addition to Rhizobiales, the orders Sphingomonadales and Rhodobacterales.

### 3.2. Bacteria Isolation and Identification

**Figure 2:**
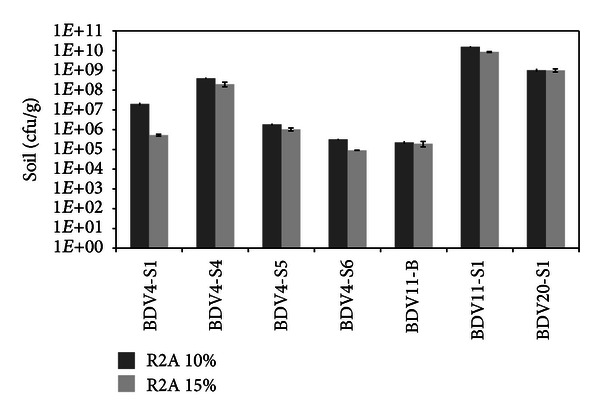
Evaluation of the halophilic/halotolerant culturable bacteria number of *Salicornia* rhizosphere and bulk soils. Microbial cell number is reported as colony-forming unit (cfu) per gram of fresh soil. Dark grey bars represent the cfu per gram of fresh soil detected on R2A medium enriched with 10% NaCl. Light grey bars represent the cfu per gram of fresh soil detected on R2A medium enriched with 15% NaCl.

Viable halotolerant bacteria were cultured on oligotrophic medium from all the collected rhizospheres and from the bulk soil sampled at BDV11 site. Culturable bacteria abundance (Figure 2) was considerably variable between the different sites, and the number of colony-forming units (cfu) ranged between 9 × 10^4^ and 1.6 × 10^10^ per gram of fresh soil. Similar counts were observed within the sites on the same medium supplemented with 10% and 15% of sodium chloride. This result indicates that most of the halotolerant culturable bacteria are able to cope with the higher value of salt content, close to the salinity measured in the soil pore water of BDV11 station. Halotolerant bacteria abundance in the BDV11-B bulk soil was in accordance with values previously reported in similar environments [40]. The interaction with the plant and the presence of root exudates could be responsible for the higher abundance of halotolerant/halophilic bacteria detected in the rhizosphere (Figure 2), as compared to the bulk soils in BDV11 site. These results are in agreement with the “rhizosphere effect” described by several authors in conventional and extreme environments [5, 39]. To our knowledge, this work is nevertheless the first report about the quantification of halotolerant microbes in root-associated extremely saline soils, enlarging the concept of the rhizosphere effect to specific bacterial groups highly adapted to the hostile environmental conditions.

**Figure 3:**
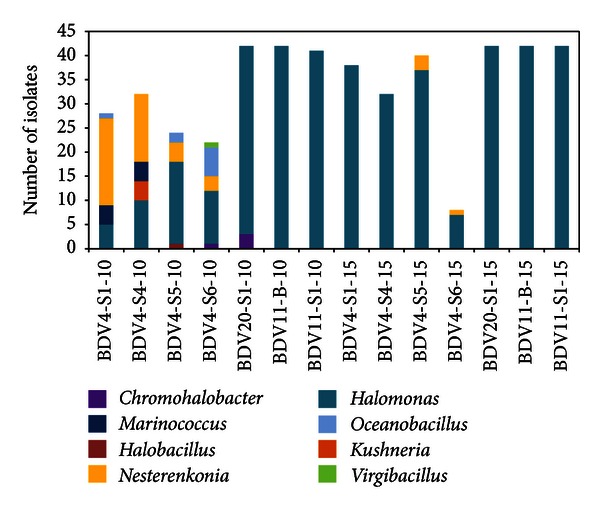
Taxonomic composition of the halophilic/halotolerant fraction of culturable bacteria associated with *Salicornia* rhizosphere and bulk soils shown as genera distribution. The numbers 10 and 15 in the sample name indicate the percentage of NaCl supplemented to the medium during isolation procedures.

A large collection of 475 isolates, representing the halotolerant culturable fraction of the bacterial diversity associated with *Salicornia *specimens and bulk soil of *Sebkhet* and *Chott* ecosystems, was established. In the case of BDV4-S6 only eight colonies growing in a medium containing 15% NaCl were isolated, whilst for the rest of the samples a number of colonies comprising between 22 and 42 were randomly picked for both the salt enrichment conditions. ITS-PCR fingerprinting was applied to dereplicate the bacteria collection allowing the identification of 136 clusters corresponding to different ITS profiles and representing different species/subspecies. From each haplotype at least one strain was arbitrarily selected for 16S rRNA partial gene sequencing and for the phenotypic tests. The taxonomic identification of the bacteria showed the prevalence of the *Halomonas* genus among the strains isolated at 15% NaCl (Figure 3). Excluding samples BDV4-S5 and BDV4-S6, where bacteria belonging to the genus *Nesterenkonia* were also retrieved, the strains isolated from both bulk and rhizosphere soils on medium containing 15% NaCl were represented exclusively by *Halomonas*. Hence, the subcollection obtained on 15% sodium chloride supplemented medium was characterized by high dominance and low values of the Shannon diversity index (Table 2). Similarly, bacteria isolated on 10% NaCl containing medium from BDV11-B and BDV11-S1 belonged exclusively to the *Halomonas* genus (Figure 3). This genus, together with the genus *Chromohalobacter*, represented the totality of BDV20-S1 halophilic culturable community (Figure 3). The prevalence of the *Halomonas* genus was demonstrated within the halotolerant root-associated bacteria collection, where *Halomonas elongata*, along with the species *H. eurihalina*, *H. sinaiensis*, *H. halmophila*, *H. ilicicola*, *H. indalina*, *H. variabilis*, *H. xinjiangensis*, and *H. taeheungii*, where retrieved. The isolation of *Halomonas* sp. from the roots of *Salicornia brachiata* was recently reported [19]. Besides harbouring higher salt tolerant bacterial counts, *Salicornia* rhizosphere was richer than bulk soils also in terms of biodiversity, since all the bacteria isolated from BDV11-B belonged to the specie *Halomonas elongata*. Overall, the strains isolated at 10% NaCl from rhizosphere soil of site BDV4 displayed a higher biodiversity at the genus level (Figure 3) that is reflected by low dominance values and high values of the Shannon diversity index (Table 2). BDV4 rhizospheric soils, characterized by lower abundance of culturable halotolerant/halophilic bacteria compared to those collected at sites BDV11 and BDV20 (Figure 2), hosted a more diverse halotolerant bacterial community comprising different genera that were previously reported in other hypersaline environments [41]. Strains belonging to the *Chromohalobacter* genus were isolated at 10% NaCl from BDV4-S6 and BDV20-S1. This genus, along with *Halomonas*, represents an important member of the family Halomonadaceae, a taxonomic group within the Gammaproteobacteria typical of hypersaline environments, whose taxonomy is still under revision [42]. A third genus, *Kushneria*, of the family Halomonadaceae was isolated from the BDV4-S4 rhizosphere. The additional root-associated halotolerant bacteria belonged to the classes Bacilli (*Halobacillus trueperi*, *Marinococcus halophilus*, *Oceanobacillus picturae* and *Virgibacillus olivae*) and Actinobacteria, the latter being represented exclusively by the species *Nesterenkonia halobia*. Both *Oceanobacillus picturae*, and *Nesterenkonia halobia* were previously isolated from a different saline ecosystem, namely, mangrove sediments [43, 44], where *O. picturea* was described for the first time as a phosphate-solubilising bacterium able to promote mangrove seedling.

Taxonomic analyses on the bacteria isolated from the rhizosphere of plants growing in different niches of the same site (BDV4-S4, BDV4-S5, and BDV4-S6) permitted to assess the occurrence of intrasite environmental selective forces shaping the composition of the culturable halophilic community, as it was already shown for the total bacterial community by DGGE-fingerprinting results. Even though the enrichment on 15% NaCl resulted in an even taxonomic distribution of the isolates, a certain degree of variability could be observed in the bacteria isolated at 10% NaCl (Figure 3), suggesting that microvariations within the site may influence the prevalence of different bacterial populations in the culturable halotolerant fraction. This result strengthens the diversity pattern described by DGGE on the total bacterial microbiome inhabiting the *Salicornia* rhizospheric soils collected at site BDV4 (Figure 1, right panel), indicating a partial overcoming of environmental factors on the rhizosphere effect imposed by the host plant.

### 3.3. Resistance to Abiotic Stresses

**Figure 4:**
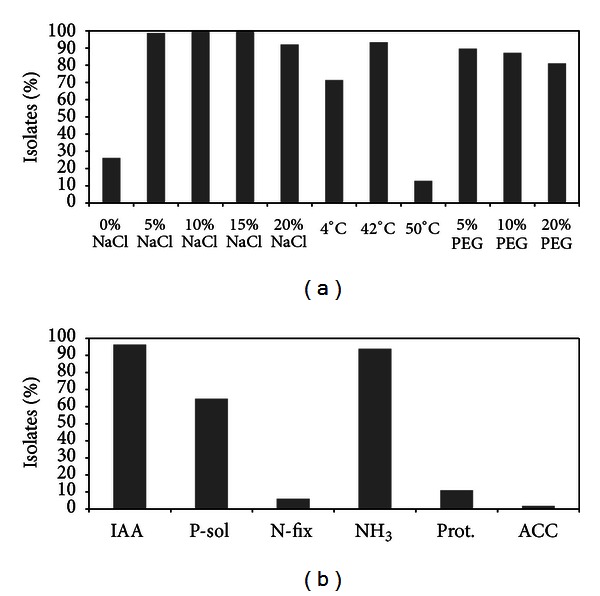
Spread of abiotic resistance and plant growth promoting traits among the halophilic/halotolerant bacteria isolated from *Salicornia* rhizosphere and bulk soils. (a) Abiotic stresses resistance. PEG: polyethylene glycol. (b) Plant growth promotion features. IAA: indole-3-acetic acid production; P-sol: phosphate solubilization; N-fix: putative nitrogen fixation ability; NH_3_: ammonia production; Prot.: Protease activity; ACC: 1-aminocyclopropane-1-carboxylate deaminase.

Aiming to identify the most suitable rhizobacteria to design a biofertilizer for sustaining plant growth in saline and arid soils, the ability of the isolated bacteria to cope with different abiotic stresses typical of arid lands was tested on 164 bacterial strains belonging to the 136 ITS groups identified by collection dereplication. In particular the bacteria collection was screened for the capability (i) to grow at extreme temperature values, (ii) to thrive in presence of different salt concentrations and (iii) in conditions of low water availability. The strains able to grow at 42°C represented 93% of the bacteria collection (Figure 4(a)) whereas only 13% of the isolates survived at higher temperature (50°C). The ability to flourish at low temperature (4°C) was observed for 71% of the total isolates, and twenty strains were able to grow in a large temperature interval, between 4 and 50°C. In arid and saline soils the vegetation is generally sparse, a factor that contributes to the strong temperature fluctuations affecting the soil. Hence, the ability of the plant associated microbes to grow in a large temperature range and to survive at temperature fluctuations is useful to efficiently colonise barren and extreme desert habitats.

All the isolates were isolated in the presence of 10 and 15% of sodium chloride in the growing medium. The majority of the isolates, corresponding, respectively, to 99% and 92% of the bacteria collection, grew at lower (5%) or higher (20%) salinity (Figure 4(a)). A significant fraction of the collection (74%) was constituted by halophiles, unable to grow in the absence of NaCl in the medium (Figure 4(a)).

A high percentage (90%) of the isolated bacteria was able to grow in presence of 5% polyethylene Glycol (PEG) (Figure 4(a)), a molecule which induces a decrease of the water potential when added to the cultivation media [45]. A decline in the number of isolates that positively grew at increasing PEG concentration was observed; nevertheless the percentage of bacteria able to grow at 10 and 20% of PEG were noteworthy and corresponded, respectively, to 87 and 81% of the bacteria collection. 

Tolerance to abiotic stresses was widespread within the bacteria collection, and represented a common trait even in phylogenetic unrelated strains, as expected since many of the retrieved species were previously isolated from saline and hypersaline habitats as in the case of *Virgibacillus* spp. [46, 47], *Halomonas sinaiensis* [48], and* Kushneria* and *Halomonas* spp. [49, 50].

*In vitro* tests showed that twenty *Halomonas* strains were particularly resistant to extreme values of different abiotic factors. These isolates, belonging to the species *H. elongata* and *H. sinaiensis*, were able to actively grow (i) on R2A medium containing a percentage of sodium chloride comprised between 5 and 20%, (ii) in the presence of 5, 10, and 20% of PEG in the medium, and (iii) when incubated in a wide range of temperature (from 4 to 50°C).

The resistance of the isolates to the extreme physical-chemical parameters of Tunisian *Sebkhet* and *Chott* ecosystems is a prerequisite to select efficient PGP bacteria able to sustain plant growth since the effectiveness of a microbial consortium strictly depends on its competitive root colonisation [51, 52], a reason that explains why the use of PGP bacteria isolated from different soil and climate conditions can be a largely unsuccessfully strategy in arid and saline lands [53, 54].

### 3.4. Plant Growth Promotion Test

The PGP activities of 164 isolates, belonging to the 136 ITS-PCR clusters and representing the whole taxonomic diversity of the established bacteria collection, were tested *in vitro* by using specific media supplemented by 5% sodium chloride.

One of the strategies adopted by PGP bacteria to induce plant growth is the influence on the plant hormonal balance. 96% of the isolates showed the ability to produce indole-3-acetic acid (IAA) (Figure 4(b)), one of the main plant hormones of the auxin family. This trait was shared by all the genera retrieved from the analysed rhizospheric and bulk soils while it was not detected in the isolated *Chromohalobacter marismortui* and *C. salexigens* strains. Few strains unable to produce IAA belong to the species *Oceanobacillus picturae* and *Halomonas halophila*, characterized by an uneven distribution of this PGP feature within their ITS clusters. The capability to modulate the plant stress level by providing indole-3-acetic acid (IAA), a molecule involved in lateral roots development, was previously reported for halotolerant bacteria isolated from coastal soils [55], halophyte roots in Argentina [56], and rhizosphere of *C. annum* growing in desert areas [5]. In addition, the recent study by Tiwari et al. [16] demonstrated that inoculation of wheat with *Halomonas* sp., the most abundant genus in our strains collection, resulted in higher content of IAA in the rhizosphere of the treated plants than control experiment.

Rhizobacteria can also positively influence the health status of the host plant by reducing the concentration of stress signaling molecule such as 1-aminocyclopropane-1-carboxylate, a precursor of ethylene. Only three strains out of the collection, belonging to the species *Halomonas taeheungii* and *Halomonas xinjiangensis*, displayed ACC-deaminase activity in presence of 5% NaCl (Figure 4(b)). ACC-deaminase activity in the genus *Halomonas* was recently reported in the ambit of the investigation of PGP features of halophilic bacteria isolated from halophytes, including the species *Salicornia brachiata* [22, 55]. Nonetheless, the low percentage (2%) of ACC-deaminase activity among the collection established in this work is in agreement with previous studies reporting the detection of ACC-deaminase activity only for a minor fraction of bacteria isolated from the rhizosphere of wheat growing in salinized soil [6, 16]. 

Direct mechanisms of plant growth promotion include those metabolisms that, by supplying nutrients to the plant, enhance its fitness. The established halotolerant/halophile bacteria collection was analysed for the capability to solubilise phosphate, fix nitrogen, and produce ammonia. The phosphate solubilisation activity was present in 65% of the whole collection (Figure 4(b)), including all the genera except for *Kushneria*. The potential activity of nitrogen fixation has been phenotypically tested by the strain capability to grow in nitrogen-free medium and confirmed by molecular investigation by PCR amplification of the *nifH* gene, codifying for a subunit of the nitrogenase enzyme. Six percent of the analysed bacterial strains were positive to both the tests showing the putative ability to fix nitrogen (Figure 4(b)). Putative nitrogen fixation activity was detected in only a minor fraction of the ITS clusters, belonging to the species *Halomonas elongata*, *H. eurihalina*, *H. indalina*, *Kushneria marisflavi*, and *Chromohalobacter canadensis*. Ammonia production was also a common PGP trait shown by 93% of the isolates (Figure 4(b)). All the bacteria genera present in strain collection were positive to the ammonia production assay, thus potentially contributing to plant nitrogen nutrition. The inability to produce ammonia did not show any species-related pattern. The widespread ability to increase the concentration of bioavailable nutrients in the isolate collection from *Salicornia* rhizosphere suggested the contribution of these halotolerant and halophilic bacteria to the plant nutrient balance. These direct PGP features were generally simultaneously present in the same strain, possibly acting in a synergic manner to directly promote plant growth, as previously reported [30].

Besides direct PGP activity, several representatives of all the taxonomic classes retrieved in our collection (11%) also displayed *in vitro* protease activity, a result that indicated their possible role as biocontrol agents. The bacterial isolates displaying protease activity comprised bacterial strains of the genera *Chromohalobacter*, *Halomonas*, *Kushneria*, *Marinococcus*, *Nesterenkonia*, and *Virgibacillus*.

The results about the investigation of the PGP traits occurrence among the bacteria collection established from the *Salicornia* rhizospheric and bulk soils are in general agreement with the observations reported by other studies realized on halophyte [22, 55, 56] and crop plant growing under saline conditions [5, 6, 15, 16].

### 3.5. *In Vitro* Colonisation of *Salicornia* Root System

**Figure 5:**
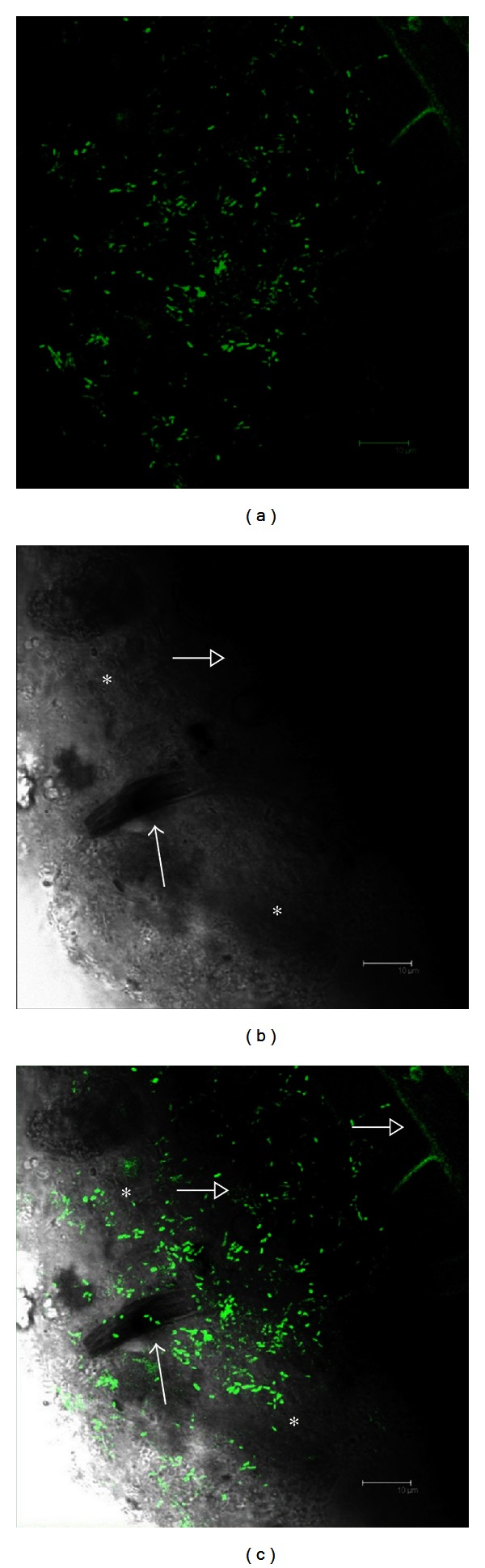
Representative images of *gfp*-tagged *Halomonas elongata* strain on *Salicornia* root acquired through BP530/30 GFP filter (excitation at 488 nm). (a) Fluorescence image showing *gfp*-*H. elongata* cells and microcolonies. (b) Bright field image of (a) showing *Salicornia* root surface (open arrow) and root hairs (arrow). (c) Overlapping of images (a) and (b) showing the colonisation of *Salicornia* root surface (open arrow in the upper right of the panel) and root hairs (arrow on the right side of the panel) by the *gfp*-tagged *H. elongata* strain. Asterisks indicated in the bright field images (a and b) show the biofilm matrix associated with the root surface. The scale bars of the images in the figure correspond to 10 *μ*m.

Besides performing *in vitro* activities involved in biostimulation, biocontrol, or biofertilization, to play an effective role in plant growth promotion, a bacterial strain should be able to colonise the plant root system. The potential ability of PGP isolates to efficiently colonise plant root system was tested by performing an adhesion assay exploiting a *gfp*-tagged PGP bacterium [57]. The adhesion test was performed on a nonhalophyte model plant, *Arabidopsis thaliana*, already used to study plant-microbe interactions [5, 58, 59], and on a wild *Salicornia* collected in southern Italy. Different rhizospheric bacterial strains belonging to the *Halomonas* genus were selected, based on their promising multiple PGP activities *in vitro* and the ability to cope with several abiotic stresses, as candidate for the chromosomal *gfp*-tagging. *H. elongata* strain BDV11S17A was successfully transformed with the gene encoding the Green Fluorescent Protein (*gfp*) that was stably inserted in the bacterium genome. The *gfp*-labelled *H. elongata* BDV11S17A was used to track bacterial adhesion on *Arabidopsis* and *Salicornia* roots *in vitro* by exposing the roots to a *gfp*-labelled bacterial suspension for 16 hours. The *gfp*-tagged strain was unable to colonise *Arabidopsis* root system, and despite several attempts and the analysis of different root specimens, only few cells were observed at the fluorescence microscope. On the contrary, confocal analysis of *Salicornia* roots showed an extensive colonisation by *gfp*-labelled strain (Figure 5). *H. elongata* BDV11S17A-*gfp*, previously shown to be able to grow under different stresses (high and low temperatures, high saline concentrations, and water stress) and to perform PGP activities *in vitro*, showed a good rhizocompetence efficiently colonizing *Salicornia* root surface and root hairs. Such features make the strain a potential candidate for *in vivo* PGP experiments.

## 4. Conclusions

DGGE fingerprinting on the total bacterial microbiome colonising the bulk soils showed that the presence/absence of salt crusts on the soil surface was a driving force involved in shaping the structure of the hypersaline soil dwelling bacterial community. The same approach demonstrated that *Salicornia* selected similar bacterial communities in the rhizosphere, independently from the site of sampling. Notably, rhizosphere associated bacterial communities differed from that colonizing the root-free soil. Overall DGGE fingerprinting indicated that a peculiar bacterial microbiome is stably associated with *Salicornia* roots, possibly having a role in promoting plant growth and stress tolerance.

The establishment of a large collection of halophilic and halotolerant bacterial strains and their identification widened the knowledge on the rhizocompetent bacterial community associated with halophytes in saline and arid soils. Furthermore, the isolation from the halophilic plant *Salicornia* of a large collection of bacteria which tolerate temperature, saline, and osmotic stresses and also showed *in vitro* the ability at medium-high salinity value (5% NaCl) to (i) positively influence the nutrients and hormonal balance and (ii) putatively express biocontrol activity as indicated by the protease activity test is a novelty presented in this study. Furthermore, the *gfp*-labelled PGP *Halomonas elongata* strain isolated from rhizospheric soil showed the ability to massively adhere on *Salicornia* roots *in vitro*, demonstrating the suitability of halophilic plants rhizobacteria to set up effective PGP inocula. The great potential of PGP halophilic and halotolerant bacteria should be carefully taken into account to satisfy the increased need of food production in the frame of a raising world population and ongoing climate changes. The present work contributes to expand the current knowledge on PGP bacteria, presenting a wide bacterial strain collection that could be exploited to set up specifically designed microbial consortia able to enhance plant growth and productivity in soils impacted by salt and drought stresses.

## Figures and Tables

**Table 1:** Sample code, location and characteristics of the *Salicornia *rhizospheres and bulk soils collected in Tunisia and analysed in the present study.

Sample code	Soil fraction	Site	Coordinates	Feature
BDV4-S1,2,3	Rhizosphere	*Sebkhet* en Naouel	N 34°26′951′′, E 09°54′102′′	soil covered by salt crust
BDV4-S4,5,6	Rhizosphere	*Sebkhet* en Naouel	N 34°26′951′′, E 09°54′102′′	soil
BDV11-S1,2,3	Rhizosphere	*Chott* El Gharsa	N 34°08′735′′, E 08°04′417′′	soil covered by salt crust (17.3 ± 1.3% of salinity)
BDV20-S1,2,3	Rhizosphere	*Chott* El Jerid	N 33°57′252′′, E 08°24′508′′	soil
BDV4-B1-1,2,3	Bulk soil	*Sebkhet* en Naouel	N 34°26′951′′, E 09°54′102′′	soil covered by salt crust
BDV4-B4-1,2,3	Bulk soil	*Sebkhet* en Naouel	N 34°26′951′′, E 09°54′102′′	soil
BDV11-B1,2,3	Bulk soil	*Chott* El Gharsa	N 34°08′735′′, E 08°04′417′′	soil covered by salt crust (19.1 ± 0.4% of salinity)
BDV20-B1,2,3	Bulk soil	*Chott* El Jerid	N 33°57′252′′, E 08°24′508′′	soil

**Table 2:** Diversity indices of halotolerant/halophilic bacteria collection. The calculation based on the genera distribution in the different analysed rhizospheric and root-free soils. The percentage indicates in brackets refers to the NaCl concentration used in the isolation medium.

	BDV4-S1 (10%)	BDV4-S1 (15%)	BDV4-S4 (10%)	BDV4-S4 (15%)	BDV4-S5 (10%)	BDV4-S5 (15%)	BDV4-S6 (10%)	BDV4-S6 (15%)	BDV20-S1 (10%)	BDV20-S1 (15%)	BDV11-B (10%)	BDV11-B (15%)	BDV11-S1 (10%)	BDV11-S1 (15%)
Genera	4	1	4	1	4	2	5	2	2	1	1	1	1	1
Individuals	28	38	32	32	24	40	22	8	42	42	42	42	42	42
Dominance	0.4668	1	0.3203	1	0.5382	0.8613	0.3471	0.7813	0.8673	1	1	1	1	1
Shannon	0.9887	0	1.245	0	0.8824	0.2664	1.254	0.3768	0.2573	0	0	0	0	0
Evenness	0.6719	1	0.8682	1	0.6042	0.6526	0.7006	0.7288	0.6467	1	1	1	1	1
